# Role of FGF Receptors and Their Pathways in Adrenocortical Tumors and Possible Therapeutic Implications

**DOI:** 10.3389/fendo.2021.795116

**Published:** 2021-12-09

**Authors:** Iuliu Sbiera, Stefan Kircher, Barbara Altieri, Kerstin Lenz, Constanze Hantel, Martin Fassnacht, Silviu Sbiera, Matthias Kroiss

**Affiliations:** ^1^ Department of Internal Medicine I, Division of Endocrinology and Diabetes, University Hospital, University of Würzburg, Würzburg, Germany; ^2^ Institute of Pathology, University of Würzburg, Würzburg, Germany; ^3^ Department of Endocrinology, Diabetology and Clinical Nutrition, University Hospital Zürich (USZ) and University of Zürich (UZH), Zürich, Switzerland; ^4^ Medizinische Klinik und Poliklinik III, University Hospital Carl Gustav Carus Dresden, Dresden, Germany; ^5^ Clinical Chemistry and Laboratory Medicine, University Hospital, University of Würzburg, Würzburg, Germany; ^6^ Comprehensive Cancer Center Mainfranken, University of Würzburg, Würzburg, Germany; ^7^ Department of Internal Medicine IV, University Hospital, Ludwig-Maximilians-Universität München, Munich, Germany

**Keywords:** normal adrenal glands, adrenocortical tumors, FGF-pathway, FGFR, RNA Expression, RNAScope, unsupervised clustering, patient survival

## Abstract

Adrenocortical carcinoma (ACC) is a rare endocrine malignancy and treatment of advanced disease is challenging. Clinical trials with multi-tyrosine kinase inhibitors in the past have yielded disappointing results. Here, we investigated fibroblast growth factor (FGF) receptors and their pathways in adrenocortical tumors as potential treatment targets. We performed real-time RT-PCR of 93 FGF pathway related genes in a cohort of 39 fresh frozen benign and malignant adrenocortical, 9 non-adrenal tissues and 4 cell lines. The expression of FGF receptors was validated in 166 formalin-fixed paraffin embedded (FFPE) tissues using RNA *in situ* hybridization (RNAscope) and correlated with clinical data. In malignant compared to benign adrenal tumors, we found significant differences in the expression of 16/94 FGF receptor pathway related genes. Genes involved in tissue differentiation and metastatic spread through epithelial to mesechymal transition were most strongly altered. The therapeutically targetable FGF receptors 1 and 4 were upregulated 4.6- and 6-fold, respectively, in malignant compared to benign adrenocortical tumors, which was confirmed by RNAscope in FFPE samples. High expression of FGFR1 and 4 was significantly associated with worse patient prognosis in univariate analysis. After multivariate adjustment for the known prognostic factors Ki-67 and ENSAT tumor stage, FGFR1 remained significantly associated with recurrence-free survival (HR=6.10, 95%CI: 1.78 – 20.86, p=0.004) and FGFR4 with overall survival (HR=3.23, 95%CI: 1.52 – 6.88, p=0.002). Collectively, our study supports a role of FGF pathways in malignant adrenocortical tumors. Quantification of FGF receptors may enable a stratification of ACC for the use of FGFR inhibitors in future clinical trials.

## Introduction

Adrenocortical carcinoma (ACC) is a rare endocrine malignancy, the pathogenesis of which is still poorly understood. Complete surgical resection is the treatment of choice in localized ACC and virtually the only option to achieve cure. As recurrence is frequent, adjuvant therapy is recommended in most patients (1, 2). The use of mitotane for adjuvant ACC treatment is mainly based on a large retrospective multicentre study conducted in Italy and Germany (3, 4). In advanced metastatic disease, the first and largest randomized phase III study in advanced ACC established etoposide, doxorubicin, cisplatin plus mitotane (EDP-M) as the cytotoxic chemotherapy of first choice (5). However, with a median overall survival of only 14.8 months in the group receiving EDP-M, the prognosis is still poor. Accordingly, there has been a growing interest in targeted therapies for the treatment of ACC.

Since insulin-like growth factor (IGF) 2 is the single most overexpressed molecule in the majority of ACC (6), clinical investigation of inhibitors of the IGF pathway yielded high expectations (7). However, this first industry-sponsored randomized phase III clinical trial in ACC with the IGF1R-inhibitor linsitinib (OSI-906) did not significantly prolong progression free survival in the vast majority of patients although some remarkable responses were observed (8). High expression of vascular endothelial growth factor (VEGF) and its receptor VEGF-R2 in many ACC specimens (9) led to several studies targeting the tumor vasculature in ACC with bevacizumab, an anti-VEGF monoclonal antibody, and sorafenib, a multi-tyrosine kinase inhibitor in combination with paclitaxel which however failed to demonstrate clinical efficacy (10). Previous *in vitro* data in ACC cell lines (11) led to the conception of a phase II clinical trial of the receptor tyrosine kinase (RTK) inhibitor sunitinib targeting VEGFR2, and PDGFRβ among others. 29 patients were evaluated in the study (SIRAC), all patients had progressed despite prior cytotoxic chemotherapy and suffered from significant tumor burden, however, sunitinib showed modest antitumor effects (12). Despite these setbacks, tyrosine kinase inhibitors have still potential in the treatment of ACC, as demonstrated by a small study using the multi-tyrosine kinase inhibitor cabozantinib in 16 patients with progressive ACC that showed prolonged disease control in half of the patients (13). However, overall, advanced disease still remains a major therapeutic challenge in patients with ACC (14).

In humans, the family of fibroblast growths factors (FGFs) comprises 22 different genes that encode proteins binding with high affinity to receptor tyrosine kinases (FGFRs) (15). Members of the FGF family function in the earliest stages of embryonic development and during organogenesis to maintain progenitor cells and mediate their growth, differentiation, survival, and patterning (16, 17). Four of the five FGF receptors (FGFR1-4) are highly conserved membrane bound RTK. After ligand binding, dimerization of the receptor causes phosphorylation of intracellular tyrosine residues that subsequently activate several crucial intracellular signaling pathways (18) like Phospholipase-C (PLC), Protein Kinase C (PKC) and Ras/Mitogen-activated protein kinase (MAPK). Activation of FGFR signaling may lead to carcinogenesis in several types of tissues (19, 20). Aberrant expression of some of the FGFs has been implicated in the development and progression of different tumor types (21, 22). Although FGF signaling can drive tumorigenesis, it has also been shown to mediate tumor protective functions (21). Importantly, the association of FGFRs with tumorigenesis led to the development of tyrosine kinase inhibitors (TKI) with FGFR specificity (23, 24), with high response rates in the first clinical studies in other types of cancer (25). Notably, response to FGFR inhibition is correlating with copy number amplification (26, 27) and mRNA expression levels (28).

The knowledge about the FGF/FGFR pathway in the adrenal gland is sparse and fragmented. As early as 1975, Gospodarowicz et al. demonstrated that some fibroblast growth factors increased proliferation in the mouse Y1-adrenocortical tumor cell line (29) and in bovine and human foetal adrenocortical cells (30). Later, FGF1 and 2 were identified as growth-stimulating factors in adrenocortical cells and adrenocortical tumors (31–33) indicating a dual role of the FGF/FGFR system in both organogenesis and tumorigenesis in the adrenal system. FGF signaling through Fgfr2 isoform IIIb was shown to regulate adrenal cortex development in mice (34) while in humans a study in 22 ACC patients has shown a variable expression of FGFR2 that did not correlate with either clinical data or CTNNB1 genotype (35). Regarding possible FGFR gene amplifications, no study is specifically reporting such data for adrenocortical tumors but a quick query in the COSMIC (https://cancer.sanger.ac.uk/cosmic) database revealed gene amplifications in FGFR1, 3 and 4 genes in 1, 4 and 3 samples in the data of Zheng et al. (36) out of 89 samples analyzed, similar to that in other types of cancer (37).

However, until now, no single study has been published that focused on the FGF/FGFR pathway as a central mechanism that can potentially be targeted therapeutically. Our study aims at closing this gap and we expect the results to represent a promising step towards a better understanding at the molecular level and improved treatment in this disease with dismal outcome.

## Material and Methods

### Patient Material

We used patient material from three normal adrenal glands (NAG), 29 adrenocortical adenomas (ACA) and 149 ACC. Of these, three NAG, 15 ACA and 21 ACC were used for the Real-time PCR experiments as frozen samples, and three NAG, 21 ACA and 142 ACC were used for RNAScope as Formalin fixed, paraffin embedded (FFPE) tissue samples (**Table 1**). For an overview of the sample overlap please see the Venn diagram in the **Supplementary Figure 1**. Informed consent was obtained from all subjects involved and the study was conducted according to the guidelines of the Declaration of Helsinki, approved by the Ethics Committee of the University of Würzburg (approval # 88/11). An overview of key clinical characteristics of the patients can be found in **Table 1**. The differential diagnosis between ACA and ACC was based on established clinical, biochemical and morphological criteria, and all histological diagnoses, including Weiss score and Ki67 expression, were confirmed by the reference pathologist. The normal adrenal glands used in this study were obtained in an anonymized fashion as a byproduct of kidney cancer surgery when the adrenal gland had to be also removed. RNA from four different ACC cell lines [NCI-H295-R, MUC-1 (38), CU-ACC1 and CU-ACC2 (39)] was included as well. The following samples served for comparison: 1 normal thyroid, 1 normal colon, 2x colon carcinoma, 2x osteosarcoma, 2x liposarcoma, 1x synovial sarcoma together with two cell lines deriving from liver cancer (Hep G2) and embryonic kidney (HEK 293).

**Table 1:** Patient and tumor statistic for the FFPE (A) and frozen tissue (B) samples.

A	NAG	ACA	ACC
*n*	3	15	21
Sex (male/female)	1/2	7/8	12/9
Age [yr (sd)]	49 (11)	46 (12)	51 (13)
Size of the tumor [cm (sd)]		3.2 (1.5)	10 (4.9)
Hormone secretion			
Cortisol – *n* (%)		8 (53%)	10 (47%)
Androgen – *n* (%)		0 (0%)	3 (14%)
Aldosterone – *n* (%)		3 (20%)	1 (5%)
Inactive – *n* (%)		4 (27%)	0 (0%)
Unknown – *n* (%)		0 (0%)	7 (34%)
Tumor localization – *n* (%)			
Primary - ENSAT stage I+II			8 (38%)
Primary - ENSAT stage III			8 (38%)
Primary - ENSAT stage IV			5 (24%)
Local recurrences			0 (0%)
Distant metastases			0 (0%)
Ki67 index [median (range)]			20 (3-70)
Weiss Score [median (range)]			7 (3-9)
**B**	**NAG**	**ACA**	**ACC**
*n*	3	29	142
Sex (male/female)	1/2	11/18	52/90
Age [yr (sd)]	49 (11)	51 (14)	49 (14)
Size of the tumor [cm (sd)]		3.3 (1.2)	9.8 (4.7)
Hormone secretion			
Cortisol – *n* (%)		11 (38%)	52 (37%)
Androgen – *n* (%)		0 (0%)	10 (7%)
Aldosterone – *n* (%)		7 (24%)	6 (4%)
Inactive – *n* (%)		11 (38%)	16 (11%)
Unknown – *n* (%)		0 (0%)	58 (41%)
Tumor localization – *n* (%)			
Primary - ENSAT stage I+II			47 (33%)
Primary - ENSAT stage III			36 (25%)
Primary - ENSAT stage IV			26 (18%)
Local recurrences			22 (16%)
Distant metastases			11 (8%)
Ki67 index [median (range)]			10 (1-70)
Weiss Score [median (range)]		0 (0-1)	5 (2-9)

NAG, normal adrenal gland; ACA, adrenocortical adenoma; ACC, adrenocortical carcinoma.

### Real-Time PCR

For quantification of mRNA expression, real-time RT-PCR was performed. RNA was extracted from previously cryo-preserved tissues using the RNeasy Lipid Tissue Kit and from cell lines using the RNeasy Mini Kit (both from Qiagen, Germany). This mRNA was then reverse transcribed with the High-Capacity cDNA Reverse Transcription Kit (Thermo Fisher Scientific, USA) and subsequently 10ng/well were used for the real-time RT-PCR amplification using the TaqMan Gene Expression Assay kit (Thermo Fisher Scientific, USA) with the probe primers for each specific gene. The pre-made FGF pathway PCR plate from Thermo Fisher (catalogue number 4418781) was utilized. The probe list and plate layout can be seen in **Supplementary Table 1**. Since the plate did not contain a probe for FGFR4, this gene was amplified separately using a specific TaqMan probe (Hs01106908_m1, Thermo Fisher), and as housekeeping genes for the expression normalization 18S (Hs99999901_s1) and ACTB (Hs99999903_m1) were amplified in parallel. The amplification and the quantification steps were performed using a StepOnePlus Real-Time PCR System (Thermo Fisher Scientific, USA). Raw expression data of the 92 genes of the FGF pathway plate were normalized to the expression of the four house keeping genes (**Supplementary Table 1**, green background) and expression of FGFR4 was normalized to the expression of the two house keeping genes listed above using the ΔCT method (Microsoft Excel). As the ribosomal 18S rRNA was one of the house keeping genes that have been used to normalize the expression data and its expression levels are much higher than the expression levels of any other gene involved in cellular function, the relative expression values resulting are expected to be quite low numerically.

### RNAScope

RNAScope is a custom RNA *in-situ* hybridization solution from Advanced Cell Diagnostics, USA. Version 2.0 of the kit was utilized for our experiments following the manufacturer’s instructions as described before (40). In short, the FFPE tissue sections of ~2µm thickness were mounted on SuperFrost glass slides (Langenbrinck, Germany), deparaffinized in xylene then washed with 100% ethanol followed by endogenous enzyme blocking in hydrogen peroxide solution (ref. part number 322335, Advanced Cell Diagnostics) at room temperature for 10 min. Permeabilization was performed by boiling in a pressure cooker for 15 minutes in target retrieval reagent (ref. 322000, ACD). Afterwards, protein digestion was achieved with the help of Protease Plus (ref. 322331, ACD) for 20 min at 40°C. All steps at 40°C were performed in a HybEZ Oven (also from ACD) that ensures quick heating up of the samples. FGFR 1, 2 and 4 probes (Hs-FGFR1 – ref. 310071; Hs-FGFR2 – ref. 311171; Hs-FGFR4-CD5 – ref. 412301, ACD) were then hybridized at 40°C for 2h. As a positive control, a probe detecting the mRNA for Cyclophilin B (PPIB) was used, which is expressed at a sufficiently low level so as to provide a rigorous control for sample quality and technical performance (ref. 313901). To ensure that there is no background staining related to the RNAscope assay a negative control probe targeting the dihydrodipicolinate reductase (DapB, accession nr. EF191515) gene from the *Bacillus subtilis* strain SMY, a soil bacterium (so unspecific for humans), was used (ref. 310043). Unbound probes were subsequently washed away. Starting with this step and until final DAB detection, the slides were washed in wash buffer (ref. 31009, ACD) instead of water. Afterwards, the slides were treated in order with Amplifier solution 1 to 6. Amplifier 1 (ref. 322311, ACD) and Amplifier 3 (ref. 22313, ACD) at 40°C for 40 min, Amplifier 2 (ref. 322312, ACD) and Amplifier 4 (ref.322314, ACD) at 40°C for 20 min, while Amplifier 5 (ref. 322315, ACD) for 50min and Amplifier 6 (ref. 322316, ACD) for 20 min at RT. Then equal amounts of DAB-A (ref. 320052, ACD) and DAB-B (ref. 320053, ACD) were mixed and applied to the slides for 10 minutes at RT. Counterstaining of nuclei was performed using Meyer’s Hematoxylin (Carl Roth, Germany) for 2 minutes, followed by washing in running tap water for 5 minutes. After dehydration, the slides were mounted using Entellan (Merck, Germany) and borosilicate glass coverslips (A. Hartenstein, Germany).

**Figure 1:**
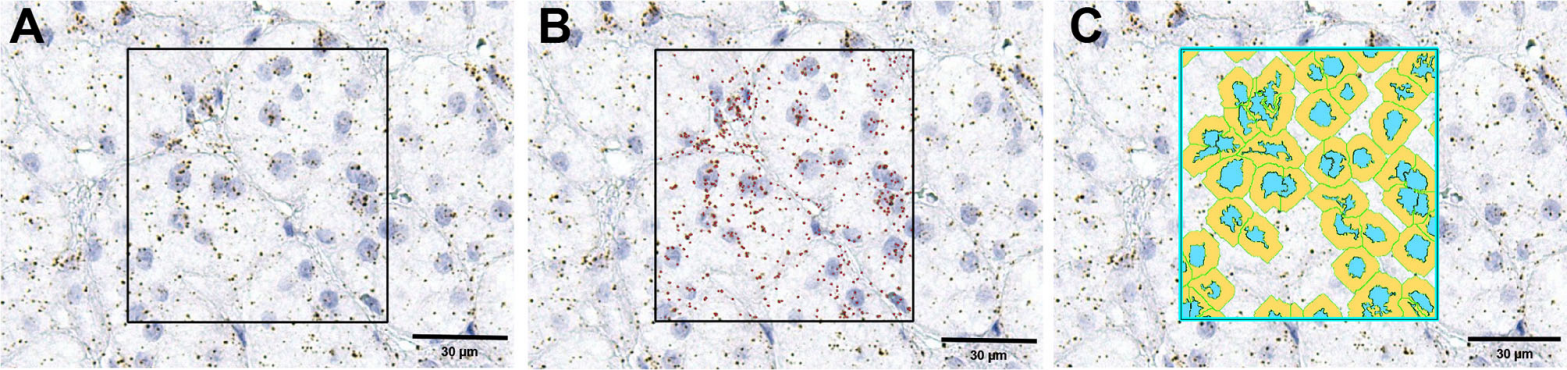
An example of RNAScope signal detection using the ImageScope software. The first image **(A)** is the original image; in the square is the selected area for detection. **(B)** the same image with the detected mRNA molecules marked in red by the software, while **(C)** is the same image with the detected cells marked in blue (nuclei) and yellow (cytoplasm).

Three pictures of representative areas of each slide were taken with the Leica Aperio Versa brightfield scanning microscope (Leica, Germany) at 40x magnification. Scoring the RNAScope slides was done with the help of Aperio ImageScope software (version 12.x, Leica, Germany) on the entirety of the pictures using the optional image analysis algorithm ‘RNA ISH v1’ (Leica, Germany). This algorithm automatically detects and counts the number of RNA molecules (each brown stained spot is one molecule of RNA) and the number of cells (by detecting the hematoxylin stained nuclei) in a defined area. Thresholds for the detection were manually adjusted for a high fidelity assessment of the signal. In **Figure 1** there is an example of only a selected area for detection from a high scoring sample. We used the ratio of RNA spots per number of cells for each slide to quantify the target gene expression.

### Bioinformatic and Statistical Analyses

Normalized expression data were Log 2 transformed and loaded into the Gene-E software (v. 3.0.215, Broad Institute ^©^2013). The unsupervised cluster analysis of all the samples was performed using the column distance/similarity matrix algorithm and the average linkage method with Pearson correlation. Hierarchical clustering of the gene expression between different phenotype clusters was performed using the marker selection algorithm, using a two-sided test with 1000 permutations. The most significantly differential expressed genes were considered those where the false discovery rate (FDR) values were <0.05.

For subsequent analyses at single gene level, non-parametrical comparisons between two groups the two-tailed Mann-Whitney test was used. A two tailed p-value <0.05 was considered statistically significant. Statistical analyses were performed with Graph Pad Prism v 7 for Windows (La Jolla, CA, USA). For ACC patients, the Kaplan-Meier method was used to estimate overall survival (OS, in all patients with primary tumors) and recurrence-free survival (RFS, in patients with complete resection of the primary tumor) while the Cox proportional hazards model was used for univariate and multivariate analyses using IBM SPSS v 26 for Windows (SPSS Inc., Chicago, IL, USA).

## Results

### FGF Pathway mRNA Expression

**Figure 2:**
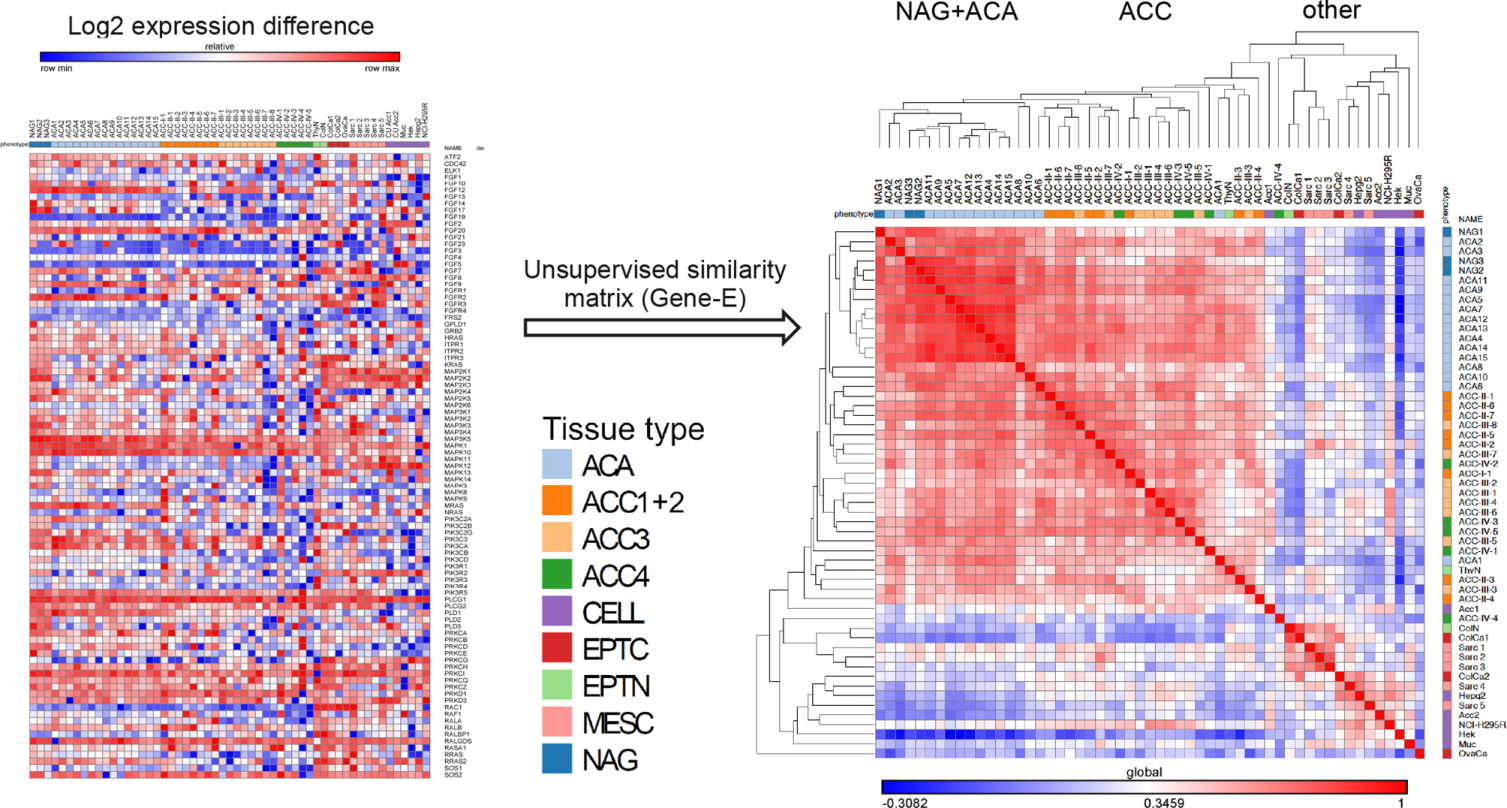
Unsupervised clustering of RT-PCR data of the FGF pathway genes. Unsupervised hierarchical matrix based on FGF pathway expression. On the left side is a graphical representation of the log 2 transformed normalized expression data arranged vertically by tissue name, and horizontally by gene name in the order that they were arranged on the PCR plate. To the right side is the data rearranged through the unsupervised similarity matrix clustering. The colored bar under the tissue names is encoding the different types of tissues analyzed: NAG=normal adrenal glands, ACA, adrenocortical adenomas; ACC1+2, ACC in ENSAT stages I and II; ACC3, ACC in ENSAT stage III; ACC4, ACC in ENSAT stage IV; EPTN, normal (non-neoplastic) classical epithelial tissues; EPTC, malignant tumors of classical epithelial tissues/carcinomas; MESC, malignant tumors of classical mesenchymal tissues/sarcomas and CELL, cell-lines.

Similarity matrix clustering of real-time RT-PCR assessment comprising 93 genes from the FGF pathway showed a distinctive phenotype of adrenocortical tissue compared to all the other tissue samples (**Figure 2**). A separate cluster that contained all three NAG and most of the ACA had a distinct expression pattern compared to the majority of ACC. At variance, typical epithelial (from thyroid and colon) and mesenchymal tissues (from sarcomas) and all cell lines showed divergent expression patterns and clustered in several small groups separately from the adrenocortical tissues. Notably, colon tissues, whether normal or malignant clustered together as did most of the sarcomas. The different cell lines, whether of adrenocortical origin or not, did not cluster with their tissue counterparts (**Figure 2**).

Several genes of the FGF pathway were significantly differentially expressed among the different clusters, including 16 genes differentially expressed between ACA and ACC (**Table 2** and **Supplementary Figure 2**). Among the 11 genes expressed at lower levels in ACC compared to ACA, there were the genes encoding for FGFs and their receptors like the *FGF12*, *FGF14*, and *FGFR2*, for phospholipases like Phospholipase D1, Phosphatidylcholine-Specific (*PLD1*) and Glycosyl-phosphatidylinositol Specific Phospholipase D1 (*GPLD1*), Ras-Related Protein R-Ras 1 (*RRAS*), 2 (*RRAS2*) and 3 (*MRAS*) and the Mitogen-Activated Protein Kinases 10 (*MAPK10*) and 5 (*MAP3K5*) as well as Phosphatidylinositol-4-Phosphate 3-Kinase Catalytic Subunit Type 2 Gamma (*PIK3C2G*). The five genes significantly upregulated in ACCs vs ACAs encoded for the *FGFR1*, *FGFR4*, *FGF8*, and *FGF19*, and the Neuroblastoma RAS Viral Oncogene Homolog (*NRAS*).

**Table 2:** Statistically significant differential mRNA expression between different groups of adrenocortical tissues.

Tissue	ACA (n = 15)	ACC (n = 21)	ACC vs. ACA	
Gene symbol	relative expression (average ± SD)	relative expression (average ± SD)	fold-change (95% CI)	*p* -value
*PLD1*	0.027 ± 0.0124	0.008 ± 0.004	-3.40 (-2.41 to -4.39)	p<0.0001
*FGF12*	0.056 ± 0.041	0.015 ± 0.024	-3.74 (-1.50 to -7.71)	p<0.0001
*RRAS2*	0.002 ± 0.0008	0.0009 ± 0.0007	-2.15 (-1.24 to -3.06)	p=0.0002
*PIK3C2G*	8.3x10^-5^ ± 1x10^-5^	1.2x10^-5^ ± 1.6x10^-5^	-6.77 (-1.70 to -11.83)	p=0.021
*MRAS*	0.025 ± 0.011	0.011 ± 0.008	-2.21 (-1.30 to -3.13)	p=0.0003
***FGFR2***	0.021 ± 0.013	0.013 ± 0.017	-1.63 (-1.03 to -2.23)	p=0.014
*MAPK10*	0.043 ± 0.028	0.021 ± 0.032	-2.00 (-1.02 to -2.98)	p=0.001
*RRAS*	0.005 ± 0.001	0.003 ± 0.002	-1.59 (-1.04 to -2.13)	p=0.006
*MAP3K5*	0.029 ± 0.018	0.011 ± 0.009	-2.52 (-1.44 to -3.59)	p=0.001
*GPLD1*	2.1x10^-4^ ± 1.3x10^-4^	1.2x10^-4^ ± 7.8x10^-5^	-1.76 (-1.15 to -2.36)	p=0.009
*FGF14*	1.1x10^-4^ ± 1.2x10^-4^	6.6x10^-5^ ± 9.8x10^-5^	-1.70 (-1.05 to -2.35)	p=0.04
***FGFR1***	0.005 ± 0.003	0.023 ± 0.015	4.11 (2.58 to 5.63)	p<0.0001
***FGFR4***	3.3x10^-5^± 2.4x10^-5^	2.0x10^-4^± 1.8x10^-4^	6.16 (3.17 to 9.14)	p<0.007
*NRAS*	0.012 ± 0.003	0.020 ± 0.009	1.45 (1.06 to 1.84)	p=0.049
*FGF8*	3.4x10^-6^± 4.7x10^-6^	2.8x10^-5^± 5.9x10^-5^	8.32 (1.26 to 15.39)	p=0.010
*FGF19*	1.7x10^-7^± 1.02x10^-7^	1.71x10^-6^± 3.0x10^-6^	9.81 (1.11 to 18.51)	p=0.047
Tissue	ACC 1 + 2 (n=8)	ACC 3 + 4 (n=13)	ACC 3 + 4 vs. ACC 1 + 2	
Gene symbol	relative expression (average ± SD)	relative expression (average ± SD)	fold-change (average ± SD)	*p* -value
*RALA*	0.016 ± 0.012	0.006 ± 0.002	-2.67 (-2.01 to -3.32)	p=0.001
*PRKCA*	0.047 ± 0.042	0.006 ± 0.006	-7.85 (-3.32 to -12.39)	p=0.004
*MAPK9*	0.031 ± 0.017	0.013 ± 0.008	-2.74 (-1.55 to -2.98)	p=0.012
*MAP3K2*	0.013 ± 0.005	0.007 ± 0.005	-1.66 (-1.04 to -2.28)	p=0.024
*PIK3R1*	0.081 ± 0.102	0.021 ± 0.019	-3.85 (-1.93 to -5.76)	p=0.024
*RAF1*	0.007 ± 0.003	0.004 ± 0.002	-1.61 (-1.11 to -2.11)	p=0.036
*MAP3K1*	0.008 ± 0.003	0.005 ± 0.002	-1.52 (-1.12 to -1.92)	p=0.036
*FGF21*	5.1x10^-7^± 5.0x10^-7^	8.8x10^-6^± 7.6x10^-6^	17.38 (9.24 to 25.51)	p=0.007

ACA, adrenocortical adenoma; ACC, adrenocortical carcinoma; ACC 1 + 2, adrenocortical carcinoma in ENSAT stages I and II; ACC 3 + 4, adrenocortical carcinoma in ENSAT stages III and IV. The higher values between two subgroups are squared in black. The significantly differentially expressed FGF- receptors are in bold type. A negative fold change is represented by the “-” sign. The p-values refer to the Mann-Whitney statistical comparison between the two groups of samples. A p-value <0.05 was considered statistically significant.

The differences between ACCs with localized, ENSAT I/II tumors compared to stage III/IV ACCs were less prominent with only 8 genes with statistically significant differential expression between the two groups (**Table 2** and **Supplementary Figure 3**). Most of these genes were expressed at lower in advanced ACC: RAS Like Proto-Oncogene A (*RALA*), Raf-1 Proto-Oncogene (*RAF1*) and the kinases Protein Kinase C Alpha (*PRKCA*), Mitogen-Activated Protein Kinase 9 (*MAPK9*), Mitogen-Activated Protein Kinase Kinase Kinase 1 (*MAP3K1*) and 2 (*MAP3K2*), and Phosphoinositide-3-Kinase Regulatory Subunit 1 (*PIK3R1*). Fibroblast growth factor 21 (*FGF21*) was the only one of the analyzed genes that was expressed at significantly higher levels in advanced ACC.

### RNAScope *In Situ* RNA Hybridization

To assess the tissue distribution of the FGF receptors *FGFR1*, *2* and *4* as potential treatment targets, and to confirm real-time PCR results in a larger sample set (n=166), we applied *RNAScope in situ RNA hybridization.*

**Figure 3:**
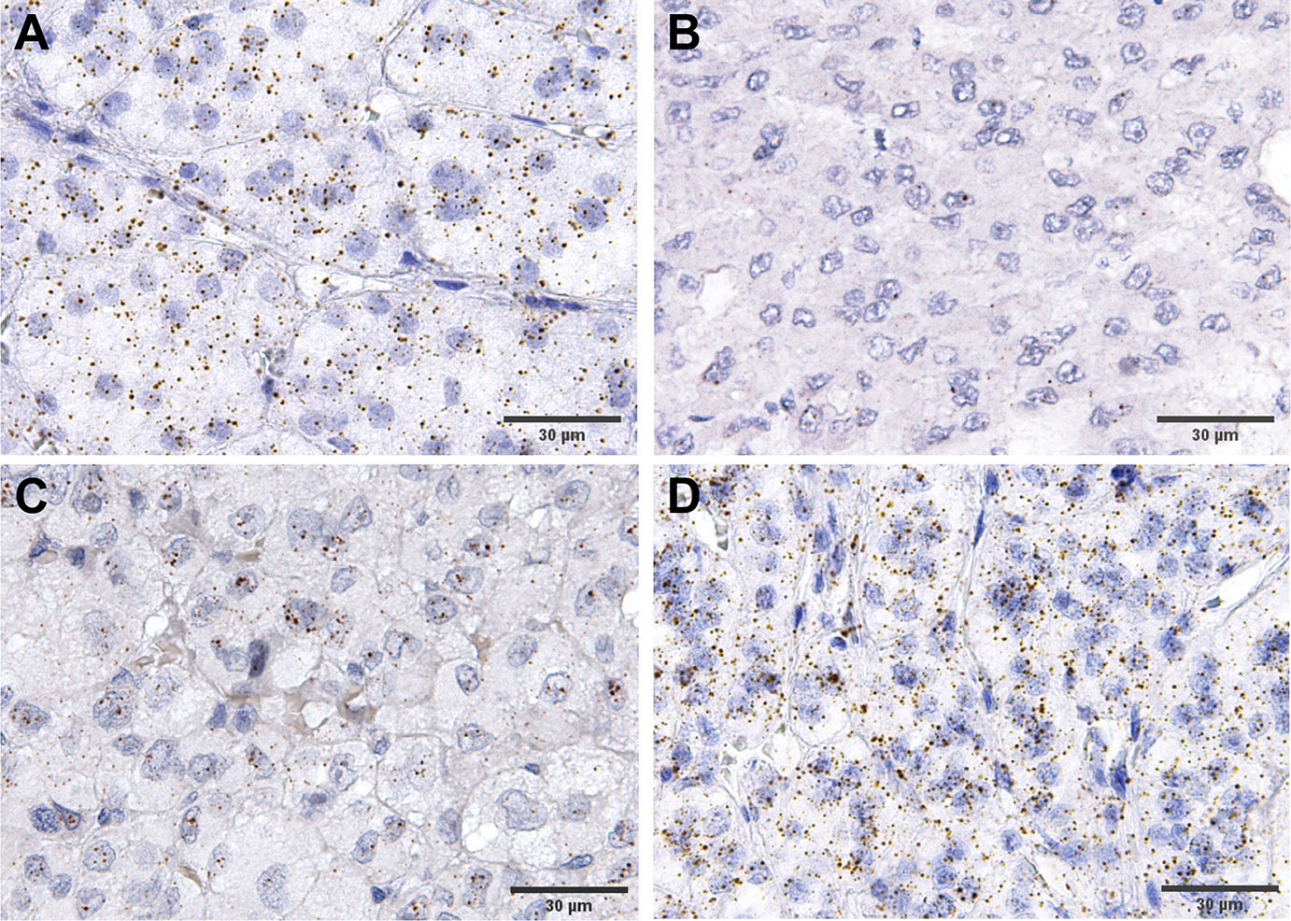
Examples of expression RNAScope staining in ACC. **(A)** an example of house keeping gene, PPIB, staining, while **(B–D)** show various levels of FGFR4 expression, from low to high.

**Figure 4:**
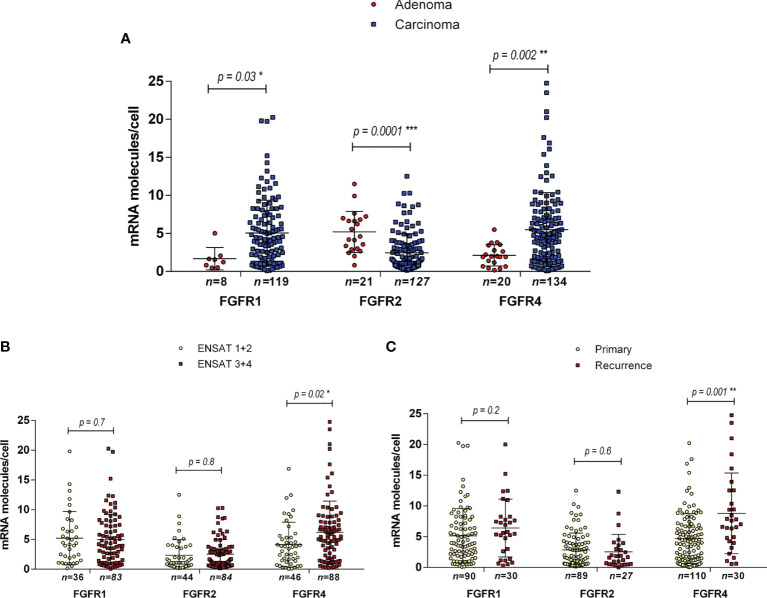
Expression of FGF receptors 1, 2 and 4 in adrenocortical tissues as assessed by RNAScope. Expression levels in ACA vs ACC **(A)**, in ACC ENSAT stages 1 + 2 vs 3 + 4 **(B)** and in ACC primary tumor samples vs local or distant recurrences **(C)**. Statistical significance: *p<0.05, **p<0.01, ***p<0.001.

We did not differentiate between the different subcellular localizations of the hybridization signal (cytoplasmic, perinuclear and nuclear) as a means to assess the complete gene specific RNA in the cells. The housekeeping gene *PPIB* (Peptidylprolyl Isomerase B) showed a relatively constant average expression in all ACC samples analyzed (between 10 and 15 molecules per cell, **Figure 3A**), and the hybridization with the bacterial DapB was negative in all the samples. The expression of *FGFRs* was variable between the samples but homogenously distributed within most samples (**Figures 3B–D**). *FGFR* expression was nearly exclusively in tumoral cells. Significantly more FGFR1 and FGFR4 mRNAs were detected in ACC compared to ACA (5.1 ± 4.3 mRNA molecules/cell vs 1.7 ± 1.4 mRNA molecules/cell, p=0.03 in the case of *FGFR1* and 5.5 ± 4.9 mRNA molecules/cell vs 2.1 ± 1.4 mRNA molecules/cell, p=0.002 in the case of *FGFR4*) (**Figure 4A**). In contrast*, FGFR2* was significantly higher expressed in ACA (5.2 ± 2.7 mRNA molecules/cell for ACA vs 2.5 ± 2.5 mRNA molecules/cell for ACC, p=0.0001) confirming our real-time RT-PCR results in frozen tissues.

Next, we compared expression between tumors in early and advanced stages and found significantly higher expression only of *FGFR4* in ENSAT stage 3 + 4 (6.2 ± 5.2 mRNA molecules/cell) compared to ENSAT 1 + 2 (4.1 ± 3.7 mRNA molecules/cell, p=0.02) ACC (**Figure 4B**). Similarly, we found significantly higher expression *FGFR4* (**Figure 4C**) in recurrences/metastasis compared to primary tumors (8.8 ± 6.6 mRNA molecules/cell for recurrences vs 4.7 ± 4.1 mRNA molecules/cell for primary tumors).

### Influence on Patient Survival

**Figure 5:**
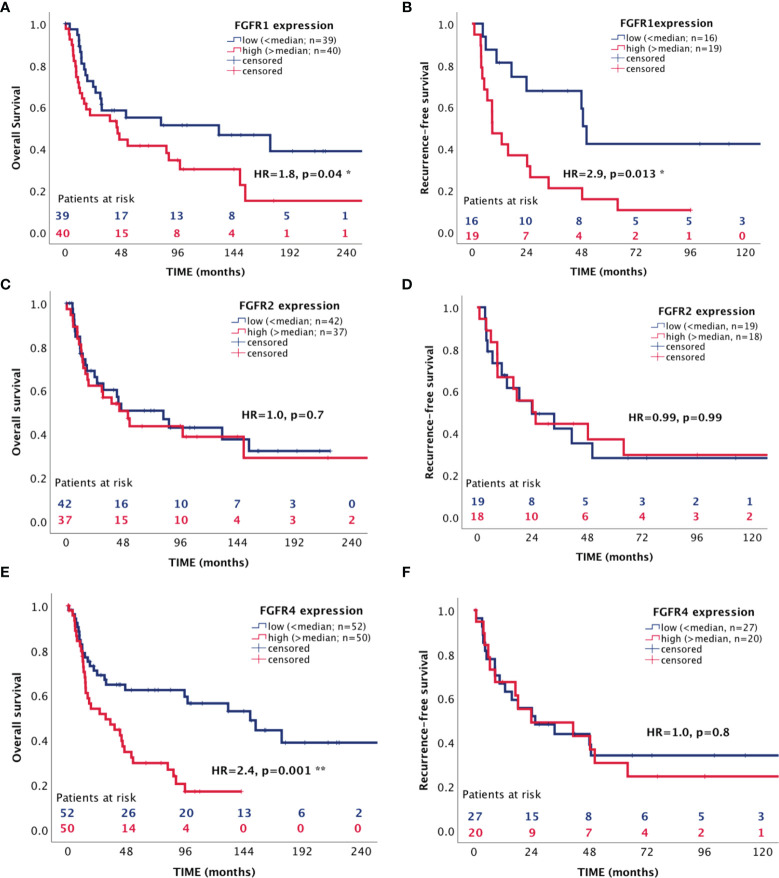
Association between expression of FGF receptors 1, 2 and 4 with patient survival. **(A, C, E)** overall survival **(B, D, F)** recurrence-free survival. *p < 0.05 and **p < 0.01.

In a next step we assessed the influence of the *FGFR 1*, *2* and *4* on patient survival in the RNAScope ACC patient cohort. The median expression value for each receptor was chosen as cut-off between low and high expression and was 3.9 spots per cell for *FGFR1*, 1.9 for *FGFR2* and 4.4 for *FGFR4*. High *FGFR1* expression was associated with both a shorter OS of 84.12 ± 16.75 *vs* 147.98 ± 23.20 months (HR=1.8, 95%CI: 1.01-3.25, p=0.047) (**Figure 5A**) and a shorter RFS of 24.84 ± 6.71 *vs* 74.71 ± 15.06 months (HR=2.93, 95%CI: 1.25-6.84, p=0.013) (**Figure 5B**), whereas *FGFR2* was not associated with either OS and RFS (**Figures 5C, D** and **Table 3**). *FGFR4*, while significantly associated with a shorter OS of 50.52 ± 7.59 *vs* 154.60 ± 19.64 months (HR=2.44, 95%CI: 1.41-4.22, p=0.001) (**Figure 5E**), was not associated with RFS (**Figure 5F** and **Table 3**). Interestingly, in a multivariate analysis, including ENSAT tumor stage and proliferation index Ki-67, two well established prognostic factors for ACC (41, 42), the association between *FGFR1* expression and the recurrence-free survival and between *FGFR4* expression and the OS remained significant (HR=6.10, 95%CI: 1.78 – 20.86, p=0.004 and HR=3.23, 95%CI: 1.52 – 6.88, p=0.002, respectively) (**Table 3**).

**Table 3:** Influence of FGFR - 1, 2 and 4 expression on overall and recurrence-free survival of ACC patients in univariate and multivariate analyses including Ki-67 and ENSAT stage.

overall survival							
Variables	univariate analysis				multivariate analysis		
	time (months)	HR	95%CI	*p*	HR	95%CI	*p*
Tumor stage (ENSAT)							
I+II (n=46)	171.68 ± 22.82						
III (n=33)	90.17 ± 17.74	2.11	1.13 – 3.94	0.018*	5.23	1.95 – 14.01	0.001**
IV (n=25)	36.94 ± 11.28	4.60	2.35 – 8.98	<0.001***	5.64	1.43 – 22.18	0.013*
Ki67							
low (<20) (n=53)	143.21 ± 17.61						
high (≥20) (n=19)	30.11 ± 6.77	4.31	2.12 – 8.78	<0.001***	17.44	5.83 – 52.20	<0.001***
FGFR1							
low (<median) (n=39)	147.9 ± 23.20						
high (>median) (n=40)	84.12 ± 16.75	1.80	1.01 – 3.25	0.047*	2.11	0.91 – 4.89	0.07
FGFR2							
low (<median) (n=42)	103.09 ± 15.94						
high (>median) (n=37)	117.81 ± 23.76	1.09	0.60 – 1.98	0.75	1.19	0.50 – 2.83	0.68
FGFR4							
low (<median) (n=50)	154.60 ± 19.64						
high (>median) (n=52)	50.52 ± 7.59	2.44	1.41 – 4.22	0.001**	3.23	1.52 – 6.88	0.002**
recurrence-free survival							
	**univariate analysis**				**multivariate analysis**		
**Variables**	**time (months)**	**HR**	**95%CI**	***p***	**HR**	**95%CI**	***p***
Tumor stage (ENSAT)							
I+II (n=35)	63.35 ± 11.58						
III (n=14)	43.65 ± 14.25	1.36	0.66 – 2.81	0.40	1.74	0.67 – 4.51	0.25
Ki67							
low (<20) (n=29)	71.22 ± 11.85						
high (≥20) (n=10)	13.89 ± 5.57	4.34	1.87 – 10.09	0.001**	8.66	2.64 – 28.44	<0.001***
FGFR1							
low (<median) (n=16)	74.71 ± 15.06						
high (>median) (n=19)	24.84 ± 6.71	2.9	1.25 – 6.84	0.009**	6.10	1.78 – 20.86	0.004**
FGFR2							
low (<median) (n=19)	59.56 ± 16.40						
high (>median) (n=18)	53.44 ± 12.71	0.99	0.45 – 2.18	0.99	0.87	0.32 – 2.39	0.79
FGFR4							
low (<median) (n=27)	62.94 ± 13.21						
high (>median) (n=20)	52.62 ± 13.07	1.06	0.52 – 2.18	0.86	0.77	0.33 – 1.80	0.55

Statistical significance:*p<0.05,**p<0.01,***p<0.001.

## Discussion

Almost half of the patients with metastatic ACC disease die of the disease already in the first year after diagnosis, one of the worst survival rates among solid cancers. This urges the need for better therapeutic options but while targeting receptor tyrosine kinases was hailed as game changing in other solid cancers, the trials with e.g. VEGFR and IGFR inhibitors in ACC were disappointing (14). This is the largest study screening the FGF/FGFR pathway in adrenocortical tissues with the declared scope of finding potential treatment targets.

The analysis of pan-FGF/FGFR pathway expression data showed that there are significant differences in the expression pattern of constituents of this pathway between the different subtypes of adrenocortical tissues but also between these and normal and neoplastic tissues of other organs. The normal and benign adrenocortical tissues clustered close together in unsupervised analyses but separately from the malignant adrenocortical carcinomas. This indicates that the different members of the pathway have similar expression patterns between the normal and benign adrenocortical tissues but different from the ACC. Interestingly, the expression in all adrenocortical tissues clustered again separately from the expression in other normal and neoplastic tissues of both epithelial and mesenchymal origin indicating that adrenocortical tissues represent a particular tissue type as we could show before (43). A defining property of FGFs and their receptors is that they bind to heparin and heparan sulfate and are therefore intimately connected with the extracellular matrix of tissues (44) where they play an important role during epithelial morphogenesis (45). As the loss of connectivity with the extracellular matrix is an important process necessary in the establishment of 2D cell-lines (46), it was not surprising that all cell-lines, including those of adrenocortical origin, had completely divergent FGF/FGFR pathway gene expression pattern from the corresponding tissues. Hence these cell-lines may not be regarded as a reliable research model for FGF signaling in adrenocortical tissues and future studies addressing the therapeutic potential of modulating these pathways will need to use more physiological models such as patient-derived tumor xenograft or spontaneous adrenocortical carcinoma mouse models.

A quantitative analysis of the genes significantly differentially expressed between the benign ACA and ACC revealed quite a high number of genes (16/93) with altered expression in ACC. A qualitative analysis of these genes showed that several of the genes that were expressed at lower levels in ACC are associated with patterns of expression indicative of tissues differentiation. Thus, a downregulation of these genes would lead to less differentiated, more disorganized tissues. For example in the adrenal, *PLD1* and *MRAS* are associated with hormonal secretion patterns (47–49), *FGFR2* is known to regulate the differentiation (50) and the spatial organization of the adrenal gland (51) while *PIK3C2G* has been identified as a novel X-zone marker and is downregulated in the adrenal glands of Gata6 knockout (cKO) mice (52). Importantly, *FGFR1* and *FGFR4* were considerably higher expressed in ACC (53–55). The concordant up-regulation of their ligands *FGF8* and *FGF19* suggests an autocrine/paracrine growth promoting loop (56).

The differences between the localized (ENSAT I and II) and more aggressive (ENSAT III and IV) ACCs were more subtle as can be observed also by the lower number of genes that had significantly different expression levels between the two subgroups. Most of the genes were associated with metastatic processes such as what was classically defined as epithelial to mesenchymal transition suggesting its involvement in the adrenocortical cancer progression. For example *RalA* plays an important role during embryogenesis and regulation of epithelial-mesenchymal interaction in tissues of mesenchymal origin including the fetal adrenal (57). The same is true for *MAPK9/JNK2* the phosphorylation status of which is controlling metastatic processes by promoting the switch of tumor cells from mesenchymal-epithelial transition to epithelial-mesenchymal transition (58) and its inactivation was identified as a carcinogenic factor in other types of cancer (59). *PIK3R1* was also reported to negatively regulate the epithelial-mesenchymal transition and stem-like phenotype of renal cancer (60) so its downregulation would lead to mobilization of cells and support establishment of metastases. Interestingly, *FGF21*, the only gene found significantly upregulated in advanced vs localized ACC is a secreted endocrine factor that functions as a major metabolic regulator stimulating the uptake of glucose and as such has been associated with aggressiveness in several types of cancer (61–63) but also with outcome in other types of diseases (64). All these findings, and especially the upregulation of the secreted factor *FGF21* in advanced ACC are important discoveries for ACC and should be addressed in more detail in further studies.

Importantly, from the therapeutic potential point of view, the three FGFRs that we could show to be differentially expressed between benign and malignant adrenocortical tumors, *FGFR1*, *2* and *4*, were also confirmed in the larger cohort of FFPE tissues. The partial cross reactivity of FGFR antibodies due to the sequence similarity of the FGFR family rendered immunohistochemistry unreliable as a validation method. That is why most studies assessing FGFR expression as a prognostic marker for selective FGFR inhibitors use an *in situ* RNA detection method instead of immunohistochemistry (28, 65, 66). We opted for the *in-situ* hybridization technique RNAScope that allowed us to both quantify and also determine the tissue distribution of the mRNA of interest, a major advantage when compared to the bulk measurement used in the targeted screening. RNAscope confirmed the previous finding that *FGFR1* and *4* are overexpressed in ACC when compared to ACA while *FGFR2* is higher expressed in the latter. Not surprisingly *FGFR1* and *4* expression was significantly negatively associated with patient survival endpoints while their individual role in recurrence and metastasis remains unclear from these clinical analyses.

The high expression of the *FGFR1* and *4* in ACCs is a promising first indication that FGFR inhibitors like ponatinib (pan-FGFR, PDGFR, SRC, RET, KIT and FLT1 inhibitor) (24), lenvatinib (VEGFR, pan-FGFR, PDGFRα, KIT and RET inhibitor) (23), rogaratinib (selective FGFR 4 inhibitor) (65) or others may have better therapeutic efficacy than the other RTK inhibitors that have been tested until now for the treatment of ACC.

To summarize our data, we could show that FGF/FGFR pathways are expressed in adrenocortical tissues and that their expression pattern is different from other tissues. Expression changes in different member molecules of this pathway are associated with tumor progression (*FGFR1* and *4*) and loss of tissue differentiation, and aggressiveness. These include factors that are generally associated with what is classically known as epithelial to mesechymal transition including cell mobilization and metastatic spread. It must be noted however from our previous work that the adrenal cortex shares less similarity with epithelial compared to mesenchymal tissues based on marker protein expression. Furthermore, *FGFR1* and *4* were also associated with patient prognosis in a relatively large cohort of ACC patients. All this data is raising the hopes that future studies with FGFR inhibitors will show the therapeutic potential of these novel targets in the treatment of refractory ACC.

## Data Availability Statement

The original contributions presented in the study are included in the article and **Supplementary Material**. Further inquiries can be directed to the corresponding authors.

## Ethics Statement

The studies involving human participants were reviewed and approved by Ethics Committee of the University of Würzburg, Josef-Schneider-Straße 4, 97080, Würzburg, Germany. The patients/participants provided their written informed consent to participate in this study.

## Author Contributions

MF, SS, and MK designed the study. IS and KL performed experiments. SK, BA, and CH provided samples and clinical data. IS and SS analysed the data. IS, MF, SS, and MK interpreted the data. IS, MF, SS, and MK wrote a manuscript. All authors contributed to the article and approved the submitted version.

## Funding

This study was supported by the Else Kröner-Fresenius Foundation (Else Kröner-Fresenius-Stiftung; project number: 2016_A96 to SS and MK and the German Research Foundation (Deutsche Forschungsgemeinschaft; project numbers 314061271 – CRC/TRR 205 and 237292849 to MF, SS and MK). This work was also supported by the Uniscientia Foundation (Keyword Tumor Model) to CH.

## Conflict of Interest

The authors declare that the research was conducted in the absence of any commercial or financial relationships that could be construed as a potential conflict of interest.

## Publisher’s Note

All claims expressed in this article are solely those of the authors and do not necessarily represent those of their affiliated organizations, or those of the publisher, the editors and the reviewers. Any product that may be evaluated in this article, or claim that may be made by its manufacturer, is not guaranteed or endorsed by the publisher.
