# Supplementary figures and images for: Role of FGF Receptors and Their Pathways in Adrenocortical Tumors and Possible Therapeutic Implications
Source: Front Endocrinol (Lausanne). 2021 Dec 9;12:795116. doi: 10.3389/fendo.2021.795116 (PMC8699171; doi:10.3389/fendo.2021.795116)

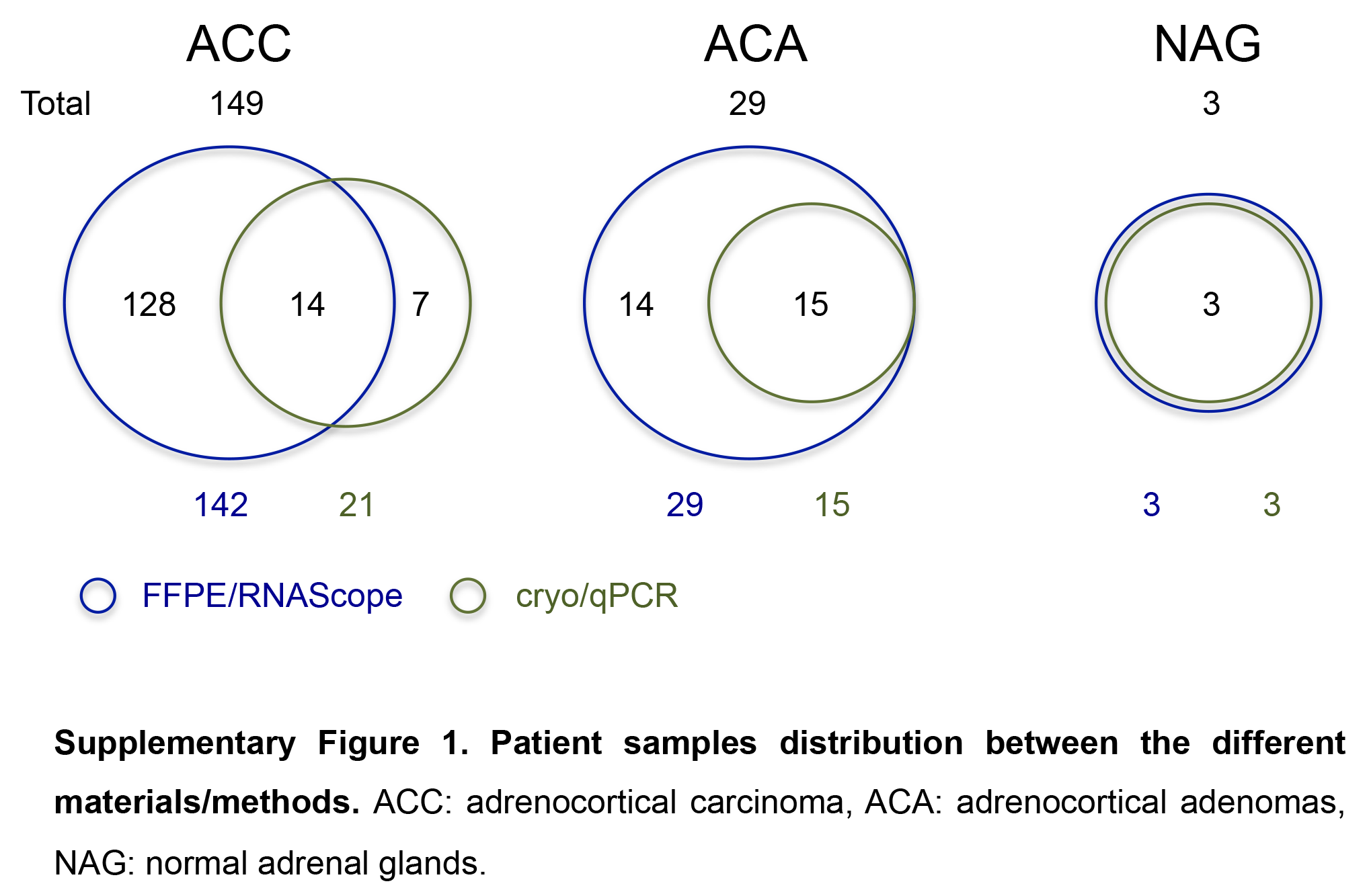

Supplement: Supplementary file 1 [file Image_1.tif]

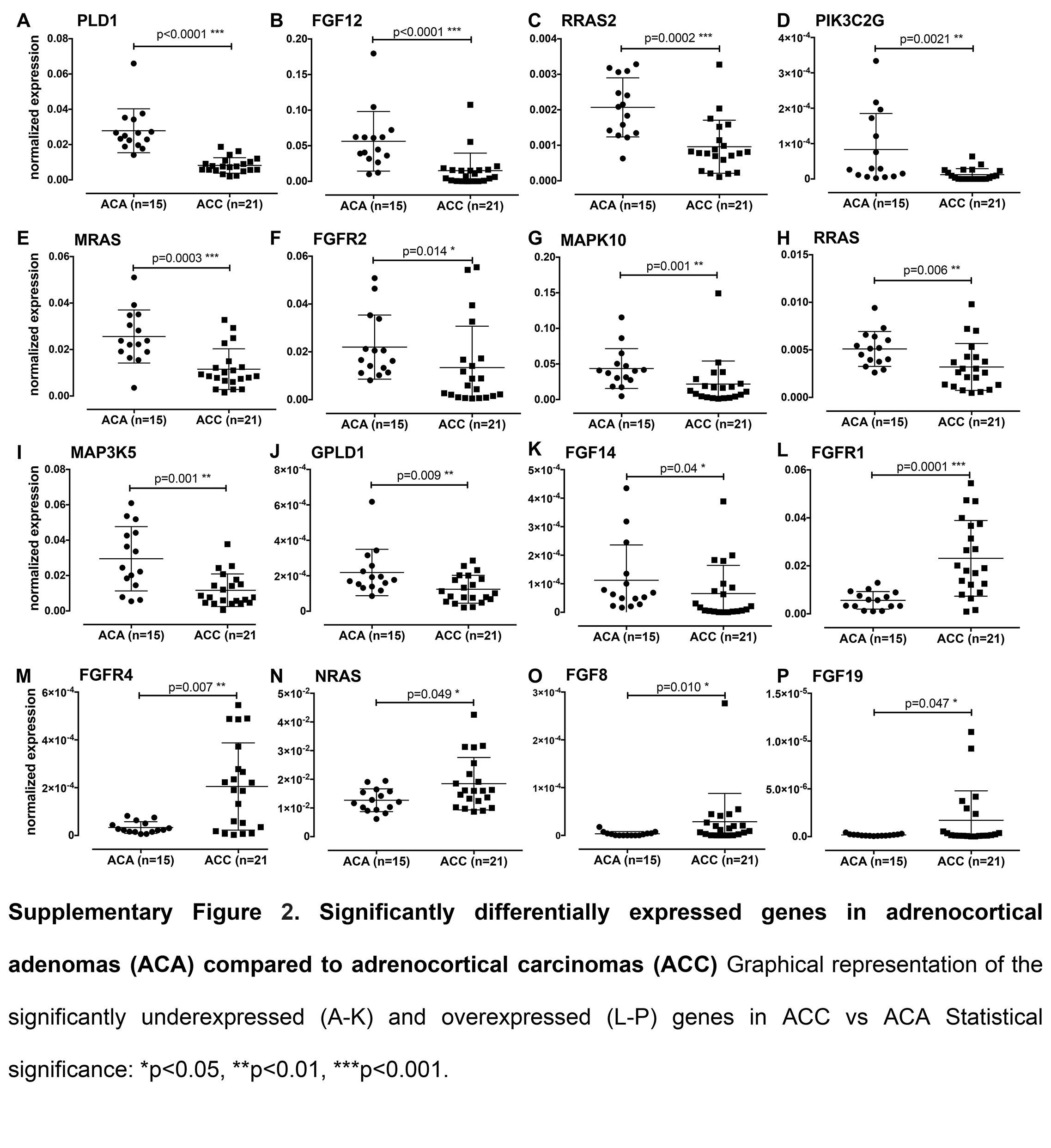

Supplement: Supplementary file 2 [file Image_2.tif]

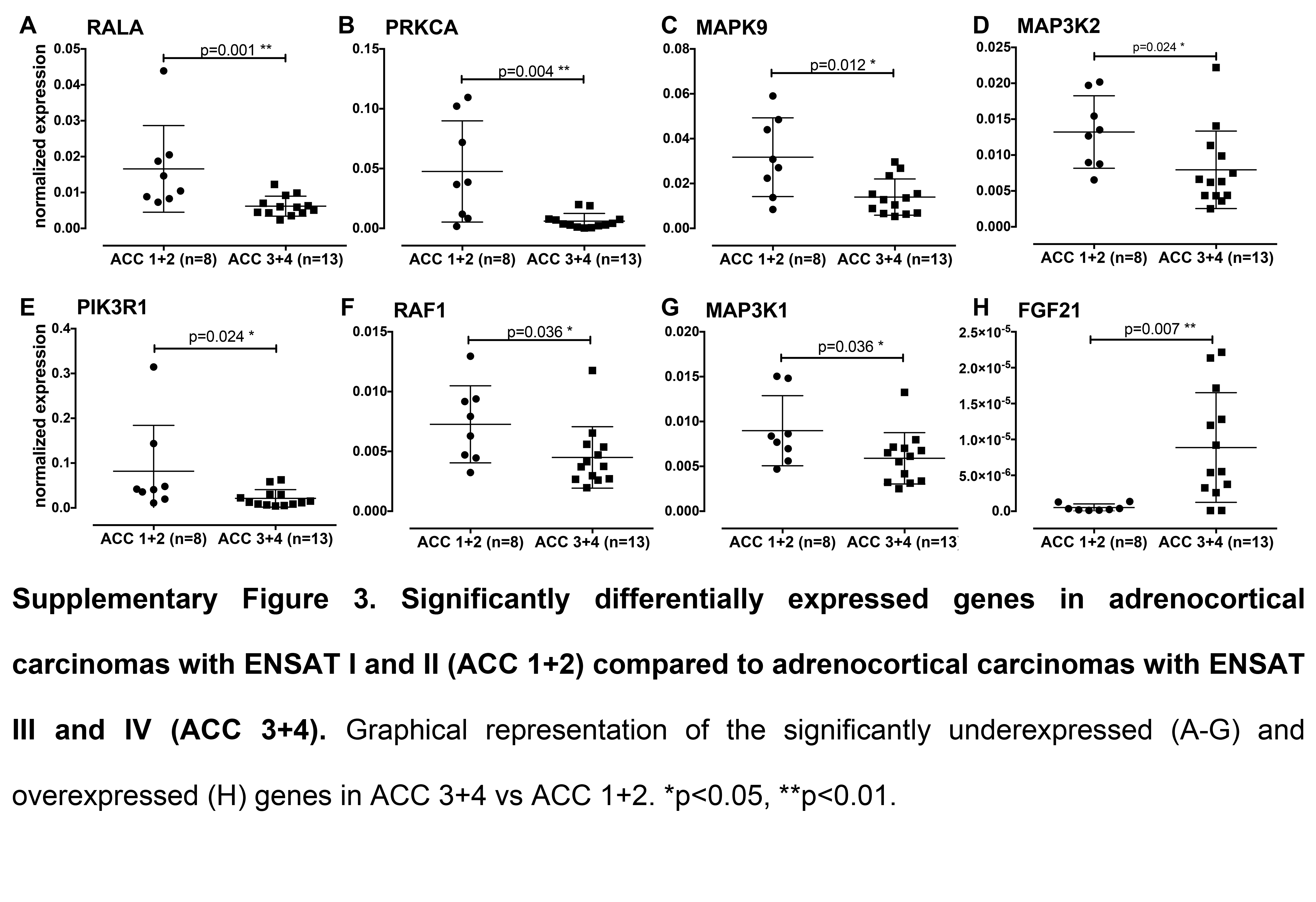

Supplement: Supplementary file 3 [file Image_3.tif]
