# Supplementary material for: Role of FGF Receptors and Their Pathways in Adrenocortical Tumors and Possible Therapeutic Implications
Source: Front Endocrinol (Lausanne). 2021 Dec 9;12:795116. doi: 10.3389/fendo.2021.795116 (PMC8699171; doi:10.3389/fendo.2021.795116)
Supplement: Supplementary file 4 [file Table_1.docx]

**Supplementary Table 1. List of all the genes being probed on the standard FGF pathway PCR plate from Thermo Fisher (Cat. nr. 4418781).**

| **Gene Symbol** | **1** | **2** | **3** | **4** | **5** | **6** | **7** | **8** | **9** | **10** | **11** | **12** |
| --- | --- | --- | --- | --- | --- | --- | --- | --- | --- | --- | --- | --- |
| **A** | **18S** | **GAPDH** | **HPRT1** | **GUSB** | **ATF2** | **CDC42** | **ELK1** | **FGF1** | **FGF10** | **FGF12** | **FGF13** | **FGF14** |
| **B** | **FGF17** | **FGF19** | **FGF2** | **FGF20** | **FGF21** | **FGF23** | **FGF3** | **FGF4** | **FGF5** | **FGF7** | **FGF8** | **FGF9** |
| **C** | **FGFR1** | **FGFR2** | **FGFR3** | **FRS2** | **GPLD1** | **GRB2** | **HRAS** | **ITPR1** | **ITPR2** | **ITPR3** | **KRAS** | **MAP2K1** |
| **D** | **MAP2K2** | **MAP2K3** | **MAP2K4** | **MAP2K5** | **MAP2K6** | **MAP3K1** | **MAP3K2** | **MAP3K3** | **MAP3K4** | **MAP3K5** | **MAPK1** | **MAPK10** |
| **E** | **MAPK11** | **MAPK12** | **MAPK13** | **MAPK14** | **MAPK3** | **MAPK8** | **MAPK9** | **MRAS** | **NRAS** | **PIK3C2A** | **PIK3C2B** | **PIK3C2G** |
| **F** | **PIK3C3** | **PIK3CA** | **PIK3CB** | **PIK3CD** | **PIK3R1** | **PIK3R2** | **PIK3R3** | **PIK3R4** | **PIK3R5** | **PLCG1** | **PLCG2** | **PLD1** |
| **G** | **PLD2** | **PLD3** | **PRKCA** | **PRKCB** | **PRKCD** | **PRKCE** | **PRKCG** | **PRKCH** | **PRKCI** | **PRKCQ** | **PRKCZ** | **PRKD1** |
| **H** | **PRKD3** | **RAC1** | **RAF1** | **RALA** | **RALB** | **RALBP1** | **RALGDS** | **RASA1** | **RRAS** | **RRAS2** | **SOS1** | **SOS2** |

The housekeeping genes for the relative quantification are marked with green background, while the FGFs and FGF receptors are marked with a red font.
